# Unlocking vegetation health: optimizing GEDI data for accurate chlorophyll content estimation

**DOI:** 10.3389/fpls.2024.1492560

**Published:** 2024-11-29

**Authors:** Cuifen Xia, Wenwu Zhou, Qingtai Shu, Zaikun Wu, Mingxing Wang, Li Xu, Zhengdao Yang, Jinge Yu, Hanyue Song, Dandan Duan

**Affiliations:** ^1^ College of Forestry, Southwest Forestry University, Kunming, China; ^2^ Guangyuan Forestry Bureau, Guangyuan, China; ^3^ School of Ecology and Applied Meteorology, Nanjing University of Information Science and Technology, Nanjing, China; ^4^ College of Forestry, Fujian Agriculture and Forestry University, Fuzhou, China; ^5^ Information Technology Research Center, Beijing Academy of Agriculture and Forestry Sciences, Beijing, China

**Keywords:** remote sensing, EBKRP method, modeling factor selection, Bayesian optimization algorithm, chlorophyll content, estimation

## Abstract

Chlorophyll content is a vital indicator for evaluating vegetation health and estimating productivity. This study addresses the issue of Global Ecosystem Dynamics Investigation (GEDI) data discreteness and explores its potential in estimating chlorophyll content. This study used the empirical Bayesian Kriging regression prediction (EBKRP) method to obtain the continuous distribution of GEDI spot parameters in an unknown space. Initially, 52 measured sample data were employed to screen the modeling parameters with the Pearson and RF methods. Next, the Bayesian optimization (BO) algorithm was applied to optimize the KNN regression model, RFR model, and Gradient Boosting Regression Tree (GBRT) model. These steps were taken to establish the most effective RS estimation model for chlorophyll content in *Dendrocalamus giganteus* (*D. giganteus*). The results showed that: (1) The *R* ^2^ of the EBKRP method was 0.34~0.99, RMSE was 0.012~3,134.005, rRMSE was 0.011~0.854, and CRPS was 965.492~1,626.887. (2) The Pearson method selects five parameters (cover, pai, fhd_normal, rv, and rx_energy_a3) with a correlation greater than 0.37. The RF method opts for five parameters (cover, fhd_normal, sensitivity, rh100, and modis_nonvegetated) with a contribution threshold greater than 5.5%. (3) The BO-GBRT model in the RF method was used as the best estimation model (*R* ^2^ = 0.86, RMSE = 0.219 g/m^2^, rRMSE = 0.167 g/m^2^, *p* = 84.13%) to estimate and map the chlorophyll content of *D. giganteus* in the study area. The distribution range is 0.20~2.50 g/m^2^. The findings aligned with the distribution of *D. giganteus* in the experimental area, indicating the reliability of estimating forest biochemical parameters using GEDI data.

## 1 Introduction

Chlorophyll content, as an important indicator of plant nutritional stress, photosynthetic capacity, growth, and senescence, plays a crucial role in assessing forest health and stand productivity (Qi and Zhang, 2016; Guo et al., 2023). *Dendrocalamus giganteus* (*D. giganteus*) is very important in maintaining the balance of forest ecosystems, addressing global warming, and contributing to carbon sequestration and emission reduction (Ni et al., 2023). The conventional approach for determining chlorophyll content is the spectrophotometer method. However, this method demands the destruction of a substantial number of plants, and there may be losses during the transportation process (Madeira et al., 2000). Moreover, it can only acquire representative sampling data from leaves, which fails to fulfill the requirements of research on the spatial distribution and variation of chlorophyll content in large-scale forests (Xu et al., 2019), thus limiting its applications in forestry. Therefore, how to efficiently, cost-effectively, and accurately estimate the chlorophyll content of *D. giganteus* at the regional level is an urgent problem that needs to be resolved in the qualitative and quantitative analysis of chlorophyll. Since the remote sensing (RS) method offers the advantages of being nondestructive, real-time, efficient, and accurate (Yao et al., 2019; Ahmad et al., 2022; Qiao et al., 2022; Wu et al., 2023), this study aims to estimate forest chlorophyll content on a regional scale using high-quality standard sampling data in combination with remote sensing data, thereby providing support for the scientific management of forest resources.

At present, the most widely used data for estimating chlorophyll content is optical RS data, such as Landsat series and Sentinel-2 data (Houborg et al., 2015; Vermote et al., 2016; Yang et al., 2023), and mainly focuses on extracting spectral features from optical RS images (Boucher et al., 2018; Cao et al., 2020; Cheng et al., 2022; Liu et al., 2024) to explore its relationship with vegetation chlorophyll content. However, research on estimating chlorophyll content using Global Ecosystem Dynamics Investigation (GEDI) data is almost blank. Compared to optical RS images, GEDI data acquisition is not limited by external environmental conditions. It has the advantages of strong penetration ability, fast capture of three-dimensional information on forest vegetation (Zhu et al., 2022; Xu et al., 2023; Zhou et al., 2024), and wide data coverage (Potapov et al., 2021; Zhou et al., 2024). In addition, the characteristic parameters such as pai, cover, and fhd_normal in the GEDI L2B product dataset have a strong sensitivity to forest biochemical parameters and forest structure parameters (Xu et al., 2023; Xia et al., 2024; Zhou et al., 2024), which is conducive to the estimation of chlorophyll content at the regional scale. However, the limitation of spaceborne LiDAR data lies in its discontinuity (i.e., the data is composed of scattered points rather than surface attribute data), leading to incomplete acquisition of forest information. In previous studies, ordinary Kriging (OK) (Zhu and Lin, 2010), inverse distance weight (Khouni et al., 2021), radial basis function (Wang et al., 2014), and other statistical spatial interpolation methods were used to solve the problem of data discreteness. However, these interpolation methods have large errors, low prediction accuracy, and unsatisfactory interpolation results. In contrast, the empirical Bayesian Kriging regression prediction (EBKRP) method can estimate the semivariogram through the process of subset and repeated simulation. While reducing the smoothing effect of spatial interpolation, it can also solve the multicollinearity problem between variables and provide a more accurate prediction of moderate non-stationary data on the local scale (Lötter and Le Maitre, 2021). This provides convenience for using GEDI spot data to obtain high-precision surface modeling feature parameters and further predict the chlorophyll content in the study area. At present, GEDI data are mostly used in the inversion of forest biomass, forest canopy closure, canopy height, etc, and it has been confirmed that the indicators in GEDI L2B product data have good model interpretation ability in estimating forest structure parameters (Potapov et al., 2021; Zhu et al., 2022; Xu et al., 2023, 2024; Zhou et al., 2024). Therefore, this study aims to explore the potential of GEDI L2B product data in forest biochemical parameter estimation.

Currently, the research on RS inversion of chlorophyll content has made great progress at home and abroad. The inversion methods mainly include the empirical model method (Shah et al., 2019), the physical model method (Darvishzadeh et al., 2012), and the coupling model method (Xu et al., 2019). The empirical modeling method (covering both parametric and non-parametric models) is primarily based on the correlation between chlorophyll content and spectral characteristics or forest structure parameters. This method is easy to operate, fast, efficient, and has ideal accuracy, but further research is needed in optimizing feature combinations. The physical modeling method is universal and does not depend on vegetation types, but it is difficult to accurately describe the radiation transfer mechanisms. Coupled models combine empirical and physical models to maximize the advantages of statistical models, but they are complex to operate and less efficient. Machine learning algorithms, as a novel modeling approach, are not constrained by fixed model frameworks and have the ability to iteratively learn from feedback errors during the model correction process, thereby enhancing the understanding of complex relationships between independent and dependent variables (Lary et al., 2016). According to previous studies, using machine learning models to estimate chlorophyll content is more accurate than using nonparametric models (Lary et al., 2016; Ta et al., 2021), but the estimation accuracy needs to be further improved. Su et al (Su et al., 2015). used a simplified support vector machine model optimized by genetic algorithm (GA-SVM) to estimate chlorophyll content. It has been proved that the optimized estimation accuracy is better than the unoptimized. At present, there are few studies on estimating chlorophyll content by the Bayesian optimization (BO) algorithm. Compared with the particle swarm optimization (PSO) algorithm, GA, and differential evolution (DE) algorithm, BO algorithm can obtain a global approximate optimal solution at a small evaluation cost (Cui and Yang, 2018), which makes the number of model optimizations less, the operation rate faster and the estimation accuracy more (Zhang et al., 2021).

Therefore, this study aims to take Xinping County, Yunnan Province, which is widely planted with *D. giganteus*, as the research area. The EBKRP method is used to obtain the attribute information of GEDI data from point to surface. The BO algorithm optimizes the machine learning model to construct the best chlorophyll content RS estimation model and evaluate the potential of GEDI data in chlorophyll content inversion. The main content of this study includes the following: (1) The construction of the chlorophyll content model of individual *D. giganteus* was realized. (2) The GEDI L2B data of spaceborne LiDAR were used as the main information source to extract the modeling parameters. Combined with 52 field-measured data, the vegetation index and terrain factor were used as explanatory variables. The empirical EBKRP method was used to obtain the continuous distribution of GEDI characteristic parameters in the unknown space of the study area. Pearson and RF methods were used to optimize the characteristic variables. Based on the BO-KNN, BO-RFR, and BO-GBRT models, the chlorophyll content of *D. giganteus* in the experimental area was established to invert the chlorophyll content and spatial distribution mapping of *D. giganteus* at the regional scale. (3) Evaluate the potential of GEDI data in estimating forest biochemical parameters, and provide a reference for the health monitoring of forest resources and the development of digital forestry.

## 2 Materials and methods

### 2.1 Study area

**Figure 1:**
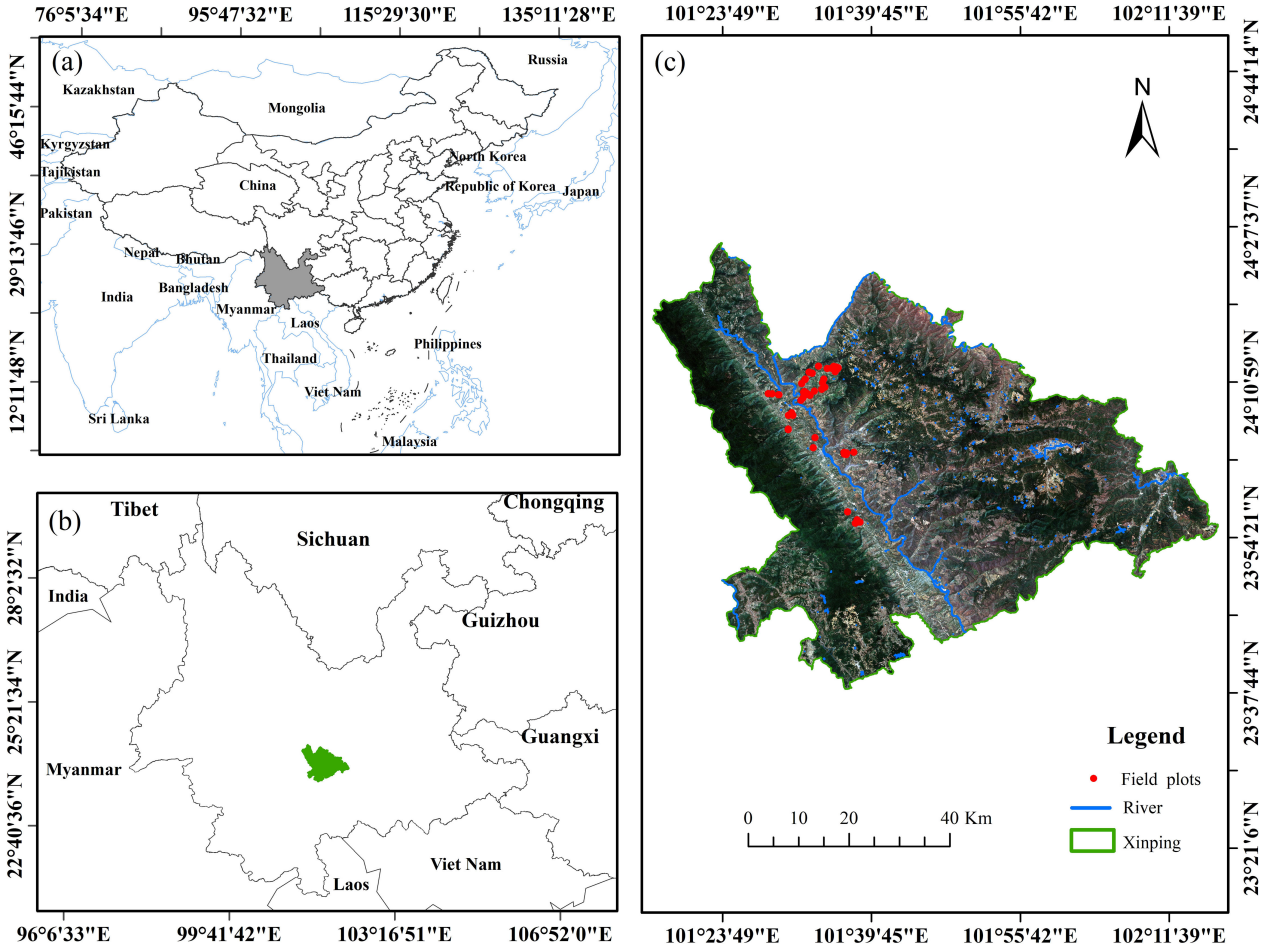
Location map of the study area (Xinping County) in China **(A)** and in Yunnan Province **(B)**. The location of the Sentinel-2 image and field sampling points in Xinping County is shown in **(C)**.

Xinping Yi and Dai Autonomous County, Yunnan Province (abbreviated as “Xinping County”), is under the jurisdiction of Yuxi City. It is located at 23°38′15″–24°26′05″ N, 101°16′30″–102°16′50″ E (**Figure 1**), with a predominantly mountainous terrain, and an elevation ranging from 422 m to 3,165.9 m. Because of the impact of altitude variation, Xinping County has formed three climate types: dry-hot valley high-temperature area, semi-mountain warm temperature area, and alpine cold temperature area. The annual rainfall is 869 mm, the annual maximum and minimum temperatures are 32.8°C and 1.3°C, respectively, and the annual average temperature is 18.1°C. It is suitable for growing in an area with an altitude of 300~1200 m, and its surface daily average temperature is required to be between 18°C and 26°C (Li, 2022). The forest coverage rate of Xinping County was 61.99%, and the forest area was 3.18 hm^2^ × 105 hm^2^. In the forest land, *D. giganteus* forest land was 1.49 hm^2^ × 104 hm^2^, accounting for 4.67%. Yunnan is one of the main distribution areas of *D. giganteus*. Bamboo forests are very important in maintaining the balance of the forest ecosystem, global warming, carbon sequestration, and emission reduction (Ni et al., 2023). The physiological and biochemical parameters of vegetation can well reflect the growth of vegetation. Therefore, accurately estimating chlorophyll content facilitates human understanding of vegetation growth and forest ecological health, promoting ecological protection, resource management, and disaster monitoring, and providing scientific basis for local governments to make informed decisions and manage forestry production precisely, thereby promoting the sustainable development of forests.

### 2.2 Ground survey data collection and processing

The 52 chlorophyll content data used in this study were collected from a standard sample plot measuring 25 m × 25 m (approximately 0.0491 km²) in the towns of Gasa, Shuitang, and Laochang in Xinping County (**Figure 1**). The number of samples saves the cost of field investigation and meets the principle of large sample field investigation (50). The rainy season in Yunnan spans from May to October, while the dry season extends from November to April. The peak rainfall occurs from mid-June to mid-August, accounting for approximately 60% of the annual precipitation. This period marks the main growth phase of *D. giganteus* and is the most representative period for reflecting changes in its chlorophyll content. Considering the comprehensive factors such as weather, road safety, representativeness and typicality of field plot setting, and stability of chlorophyll content of *D. giganteus*, it was found that the weather was sunny from 4 to 15 January. Therefore, from 7 to 14 January, the field survey was carried out in the study area to collect experimental data. The diameter at breast height, coordinates, and other factors were measured, and the materials required for the experiment (including standard *D. giganteus* and standard leaves) were collected. Among them is the diameter of the diameter at breast height (DBH, namely the diameter at the position between the root collar and breast height (Li, 2019). There are differences among various countries in the regulation of breast −height position. In China and continental Europe, it is approximately 1.3 m. In the UK, it is around 1.32 m. In the USA and Canada, it is about 1.37 m, and in Japan, it is 1.2 m.) is set to 5 cm, and the *D. giganteus* with a DBH of 10 cm is used as the standard plant. The coordinates of each sample plot were measured using a differential locator in the fixed solution state of the southern surveying and mapping real-time kinematic (RTK), with an error margin of less than 2 cm.

#### 2.2.1 *D. giganteus* standard selection and sampling method

In 52 sample circles, different age classes of *D. giganteus* with no pests and diseases, no mechanical damage, and healthy growth were randomly selected as standard samples, totaling 141 *D. giganteus* plants. The DBH was measured from the base, and all fresh leaves of 141 *D. giganteus* were collected and weighed (**Table 1**).

**Table 1:** Statistical table of measuring index of single *Dendrocalamus giganteus* plant.

Name	Sample size	Maximum	Minimum	Average	SD
DBH (cm)	141	12.8	3.2	8.5	2.3
Total fresh weight of leaves (kg)	141	6.54	0.05	1.91	1.32

#### 2.2.2 Selection of standard leaves and determination of samples

Forty-nine standard *D. giganteus* plants were randomly selected, and leaves of varying sizes—new, old, and young—were collected from the upper, middle, and lower sections of each plant to serve as standard sample leaves. The determination of chlorophyll content involves three main steps: first, the surface of the standard leaves was cleaned, and samples were evenly collected from the upper, middle, and lower parts on both sides of the central vein. A 0.2-g fresh sample was then weighed. In the second step, the weighed sample was cut into pieces and placed in a mortar. Liquid nitrogen was added to powder the sample, followed by the addition of 80% acetone for grinding into a homogenate, which was then filtered and brought to a constant volume. In the third step, the chloroplast pigment extract was poured into a colorimetric dish with a light path of 1 cm, using 80% acetone as a blank control. The absorbance was then measured using a spectrophotometer at 663 nm and 645 nm. The results are presented in **Table 2**. The specific method for the determination of chlorophyll in the standard leaves of *D. giganteus* refers to the plant’s physiological and biochemical experiment principle and technology (Wang and Huang, 2015), and the calculation formula is as follows:

**Table 2:** Statistical table of chlorophyll content of *D. giganteus* leaves.

*Chl* (mg/g)	Sample size	Maximum	Minimum	Average	SD
*C_a_*	49	2.67	1.78	2.36	0.26
*C_b_*	49	2.73	0.35	1.27	0.54
*C_t_*	49	5.34	2.13	3.63	0.78


$$\begin{array}{c} {\text{C}}_{\text{a}}=12.72{\text{β}}_{663}-2.59{\text{β}}_{645} \end{array}$$

$${\text{C}}_{\text{b}}=22.88{\text{β}}_{645}-4.67{\text{β}}_{663}$$

$${\text{C}}_{\text{t}}={\text{C}}_{\text{a}}+{\text{C}}_{\text{b}}=20.29{\text{β}}_{645}+8.05{\text{β}}_{663}$$

where *C_a_*, *C_b_*, and *C_t_* represent the contents of chlorophyll *a*, chlorophyll *b*, and total chlorophyll, respectively (in units of mg/g). $\beta_{663}$ and $\beta_{645}$ denote the absorbance of the chloroplast pigment extract at wavelengths of 663 nm and 645 nm, respectively.

#### 2.2.3 Measurement of chlorophyll of *D. giganteus* at plot scale

Based on the allometric growth equation, the chlorophyll content and DBH of 141 individual *D. giganteus* were used as dependent and independent variables, respectively, to construct a basic model for predicting the chlorophyll content of individual *D. giganteus*. Using this model, the chlorophyll content of 52 sample plots was calculated (**Table 3**).

**Table 3:** Statistical table of chlorophyll content in *D. giganteus* sample circles.

Name	Sample size	Maximum	Minimum	Average	SD
*Chl_T_* (g/m²)	52	2.74	0.17	1.32	0.57

### 2.3 Acquiring and extracting information from RS data

#### 2.3.1 GEDI data

The GEDI is a new multibeam full-waveform LiDAR sensor (Liang et al., 2023; Xu et al., 2023). It is mounted on the United States International Space Station (ISS) and was successfully launched by NASA on 5 December 2018 at the Kennedy Space Center in the USA. The sampling collection encompasses data from latitudes of 51.6° North to 51.6° South worldwide. The GEDI employs three lasers, which produce a total of eight beams. Each footprint sample measures approximately 25 m, with an orbital interval of about 60 m. The beams are spaced approximately 600 m apart in the cross-orbit direction. Additionally, the cross-track width is around 4.2 km. The GEDI comprises four product-level datasets: L1 is geo-referenced return energy waveform data, L2 provides georeferenced surface elevation and canopy height, L3 offers gridded vegetation structure, and L4 includes footprint-level and gridded aboveground biomass data (Xia et al., 2024).

This study utilizes L2B data, which provides more comprehensive information than L2A data, including variables such as cover, pai, and fhd_normal (Crockett et al., 2023; Xu et al., 2023; Zhou et al., 2024). The GEDI data utilized in this study was downloaded from the Earthdata website (https://search.earthdata.nasa.gov/) in March 2024. The selection process focused on beam data that covered Xinping County from 1 January 2022 to 31 March 2023. This resulted in a total of 41 orbit datasets, comprising 164 orbits and 328 orbit beams. To ensure the acquisition of high-quality footprint points, we filtered out invalid light spots, drawing on methodologies from previous studies (**Table 4**) (Liang et al., 2023; Xu et al., 2023; Xia et al., 2024; Zhou et al., 2024).

**Table 4:** GEDI footprint quality filtering criteria.

Parameters	Retention value	Retention basis
lon_lowestmode	101°–103° E	The geographical longitude range of Xinping County.
lat_lowestmode	23°–25° N	The geographical latitude range of Xinping County.
quality_flag	1	Indicates good quality of the footprint spots.
Sensitivity	≥ 0.90	Close to 1, good quality.
degrade_flag	0	Indicates good data performance.

**Figure 2:**
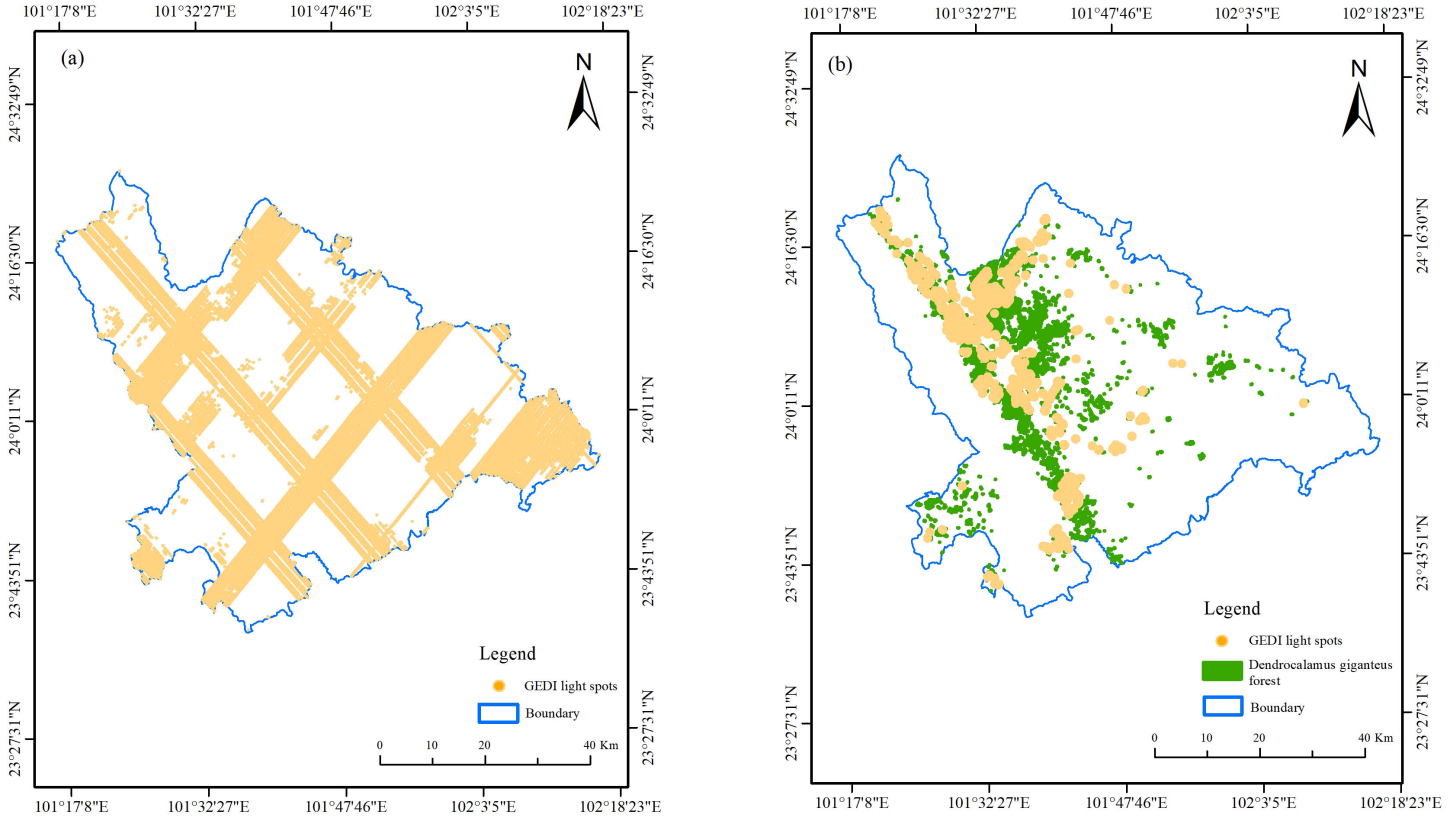
**(A)** Distribution of all spots. **(B)** Distribution of light spots in *D. giganteus* forest.

After screening out the target points through the above five indicators, 51,669 effective footprint points were obtained in the study area, and then the spatial overlay analysis of the light spots in the *D. giganteus* forest area was carried out using the 2016 Forest Resources Survey data. There are 1,670 spots in the *D. giganteus* forest land and 49,999 spots in the non-*D. giganteus* forest land, as shown in **Figure 2**. The meaning of the GEDI feature parameters extracted in this study can be obtained by searching the “GEDI L2B Product Data Dictionary” through the browser, which covers 31 modeling alternative feature variables and five quality screening parameters used in the study.

#### 2.3.2 Sentinel-2 data

Sentinel-2 is the second component of the European Space Agency (ESA) Copernicus series of satellites. It consists of two complementary satellites, satellites 2A and 2B. The satellite belongs to the medium-resolution multispectral optical imaging satellite. The revisit period is 6 days. The spectrum obtained on the sensor contains 13 bands. The spectral range is 0.4~2.4 µm, covering visible light, near-infrared, short-wave infrared, and other image data in different wavelength ranges. The ground resolutions are 10 m (bands B2, B3, B4, and B8), 20 m (bands B5, B6, B7, B8a, B11, and B12), and 60 m (bands B1, B9, and B10) (Phiri et al., 2020). In this study, 2A-level product data were used. The data were downloaded from the official website of Google Earth Engine (GEE) dataset (https://developers.google.com/earth-engine/datasets) for free. The access date was April 2024. The data acquisition time range was from 1 May 2023 to 14 January 2024. The cloud amount was set to ≤ 5%, and the resolution was set to 25 m. After downloading, the software ENVI 5.6 was used to extract 12 factors such as vegetation index (Ma et al., 2021) and topographic features (extracted from 25 m DEM after resampling) (Wu et al., 2016) as explanatory variables of EBKRP (**Table 5**).

**Table 5:** Lis of Sentinel-2 data RS factor information extraction.

Variables	Amount	Description
DVI	1	Difference vegetation index: $DVI=\rho_{NIR}-\rho_{R}$ , $\rho_{NIR}$ , $\rho_{R}$ , the reflectance of the near-infrared band and the red band, respectively.
RVI	1	Ratio vegetation index: $RVI=\rho_{NIR}/\rho_{R}$ .
NDVI	1	Normalized vegetation index: $NDVI=\left(\rho_{NIR}-\rho_{R}\right)/\left(\rho_{NIR}+\rho_{R}\right)$
SAVI	1	Soil-adjusted vegetation index: $SAVI=\frac{1.5\left(\rho_{NIR}-\rho_{R}\right)}{\rho_{NIR}+\rho_{R}+0.5}$
EVI	1	Enhanced vegetation index $EVI=2.5\left[\frac{\left(\rho_{NIR}-\rho_{R}\right)}{\left(\rho_{NIR}+6.0\rho_{R}-7.5\rho_{B}+1\right)}\right]$ , $\rho_{B}$ is the reflectivity of the blue band.
NPCI	1	Normalized pigment chlorophyll index: $NPCI=\left(\rho_{R}-\rho_{G}\right)/\left(\rho_{R}+\rho_{G}\right)$ , $\rho_{G}$ is the reflectivity of the green band.
GNDVI	1	Green normalized difference vegetation index: $GNDVI=\left(\rho_{NIR}-\rho_{G}\right)/\left(\rho_{NIR}+\rho_{G}\right)$
GRVI	1	Green ratio vegetation index: $GRVI=\rho_{NIR}/\rho_{G}$
GDVI	1	Green difference vegetation index: $GDVI=\rho_{NIR}-\rho_{G}$
Elevation	1	Elevation
Slope	1	Slope factor extracted by DEM
Aspect	1	Slope aspect factor extracted by DEM

The 12.5-m DEM data used in this study were derived from the polarimetric synthetic aperture radar (PALSAR) sensor of the Advanced Land Observing Satellite (ALOS). The data were downloaded from the Earthdata website (https://search.earthdata.nasa.gov/search), and the access date was April 2024. After download, it was resampled to 25 m × 25 m using the resampling tool under the geostatistical software ArcGIS 10.8 to match it with the plot size. In addition, this study also used the subcompartment data of the forest resources survey in the study area in 2016, which was used for mask extraction of *D. giganteus* forest land.

### 2.4 Research method

**Figure 3:**
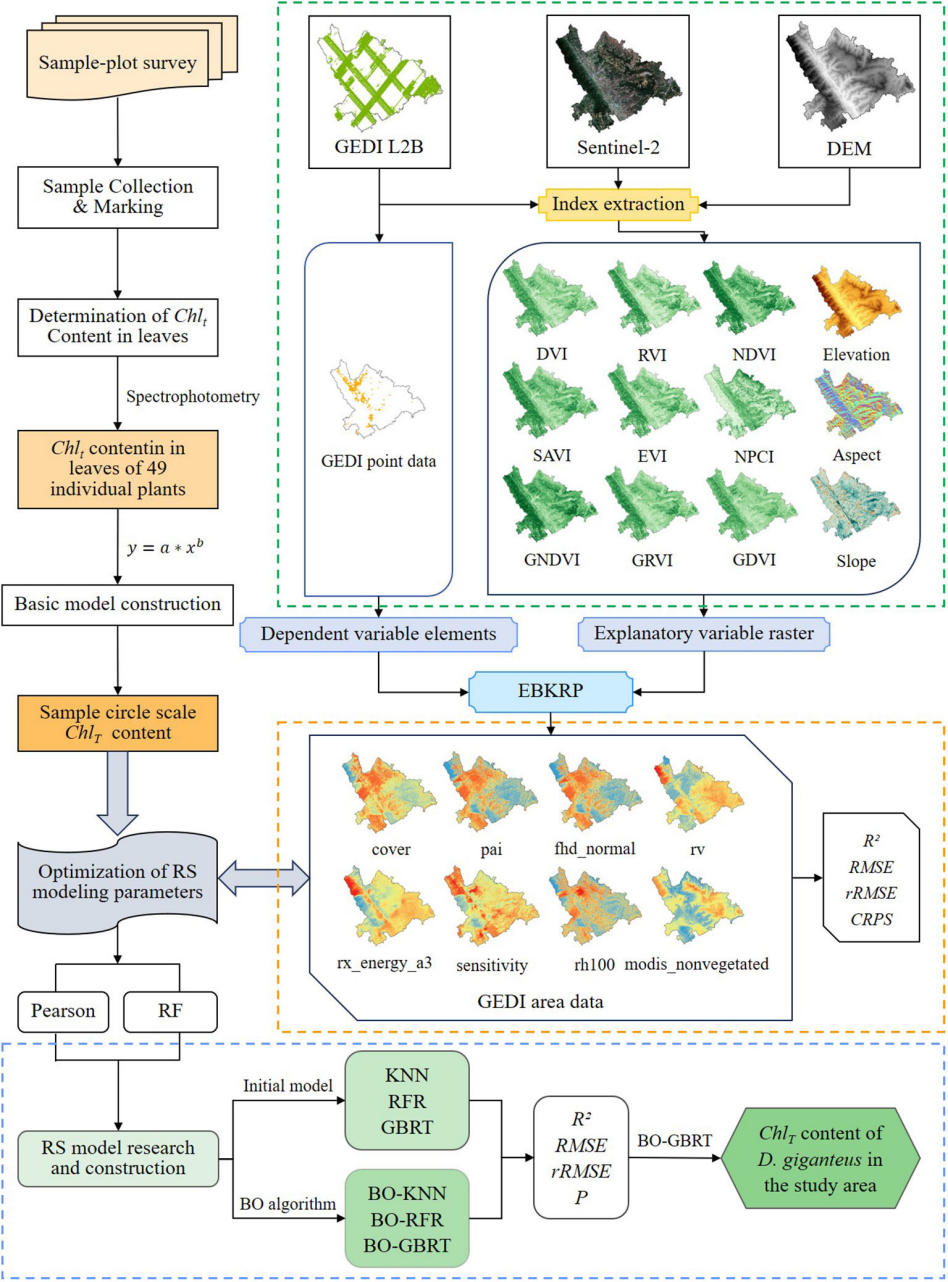
Technical route.

The process for estimating and inverting the chlorophyll content of *D. giganteus* at a regional scale using the BO algorithm involves several key steps: (1) First, constructing a basic model for the chlorophyll content of individual *D. giganteus*; (2) Next, selecting the Kriging regression method and assessing its accuracy; and (3) finally, optimizing the RS modeling parameters are optimized for regional-scale chlorophyll content estimation. This optimization process utilizes the basic model to calculate chlorophyll content at the sample plot level. The calculated values serve as the training samples (dependent variables) for RS modeling, while characteristic values of feature variable factors, extracted from corresponding sample plot points by GEDI L2B, are used as modeling samples (independent variables). This approach facilitates the construction of an optimal predictive model for *D. giganteus* chlorophyll content at a regional scale. Subsequently, this model is applied to invert the chlorophyll content of *D. giganteus* in Xinping County (**Figure 3**).

#### 2.4.1 Construction of the basic model for the chlorophyll content of individual *D. giganteus*

This study utilized the allometric growth equation to investigate the potential of GEDI parameters for directly estimating the chlorophyll content of *D. giganteus* at a regional scale. By destroying a small number of limited samples, a power function relationship between chlorophyll content and the DBH of a single plant (Wang et al., 2021) was established as the basic model of chlorophyll content of a single plant. The chlorophyll content of 52 sample circles was then calculated using the following formula.


$$\text{C}h{\text{l}}_{\text{T}\;}=\text{a}D\text{B}{\text{H}}^{b}$$

Where $Chl_{T}$ denotes the chlorophyll content of an individual *D. giganteus* plant, while DBH refers to its diameter. The parameters a and b are those to be estimated in the model for a single plant.

#### 2.4.2 Geostatistics method

##### 2.4.2.1 EBKRP method

The EBKRP method is a geostatistical spatial regression method combining least squares regression and the OK method. The data are processed by the geostatistical software ArcGISpro 2.8, and the known sample data in the test area is used to estimate the unmeasured data in the test area, and then the contour map of GEDI parameters and the area attribute data map predicted by EBKRP are generated (Goovaerts, 1997; Chilès and Delfiner, 2012). The transformation of GEDI data from point to surface enables the extraction of regionalized variables for constructing functional relationships with chlorophyll content, thereby allowing for the determination of chlorophyll content’s spatial distribution characteristics (Curran and Atkinson, 1998). The main steps include (1) preparing sampling light spot attribute data (GEDI) and explanatory variable grid attribute data (Sentinel-2 and DEM) to enhance the relationship with chlorophyll content and improve the prediction accuracy; (2) constructing the variogram, setting main variogram parameters (such as nugget value) or selecting models (such as index, subtraction function, or K-Bessel); (3) assessing the anisotropy of Kriging estimation, as spatial correlation typically depends on both the distance between points and the direction of sampling; and (4) establishing the experimental variance diagram and testing the cross-validation results.

##### 2.4.2.2 Accuracy evaluation of EBKRP

For the regression results and fitting accuracy of the EBKRP method, this study uses cross-validation in geostatistics to evaluate its accuracy. The process of this verification method is to remove a point in the dataset, use the remaining points to predict the position of the removed point, and compare the measured value with the predicted value to determine the accuracy of the prediction (Zhou et al., 2023, 2024). The determination coefficient (*R*-squared [*R* ^2^]), root mean square error (RMSE), relative root mean square error (rRMSE), and continuous ranked probability score (CRPS) were utilized in this study as the comprehensive evaluation indexes for the EBKRP method. A model’s goodness of fit improves as the *R* ^2^ value approaches 1. Greater model accuracy is indicated by lower values of RMSE and rRMSE. A model’s performance is deemed better when the CRPS value is closer to 0. Conversely, the regression performance of the GEDI parameter space was found to be less than optimal. The definition formula of each evaluation index:


$${\text{R}}^{2}=\frac{{\sum_{\text{i}=1}^{\text{n}}\left[\hat{\text{Z}}({\text{x}}_{\text{i}})-\bar{\text{Z}}({\text{x}}_{\text{i}})\right]}^{2}}{{\sum_{\text{i}=1}^{\text{N}}\left[\text{Z}({\text{x}}_{\text{i}})-\bar{\text{Z}}({\text{x}}_{\text{i}})\right]}^{2}}$$

$$\text{RMSE}=\sqrt{\frac{{\sum_{\text{i}=1}^{\text{n}}\left[\text{Z}({\text{x}}_{\text{i}})-\hat{\text{Z}}({\text{x}}_{\text{i}})\right]}^{2}}{\text{n}}}$$

$$\text{rRMSE}=\frac{\text{RMSE}}{\bar{\text{Z}}({\text{x}}_{\text{i}})}$$

$$\text{CRPS}(\text{A},\text{F})={\int \left[\text{F}(\text{x})-1\{\text{x}\geq \text{y}\}\right]}^{2}\text{d}x$$

Where $\overset{\wedge }{Z}(x_{i})$ is the predicted value of x at i position, $\bar{Z}\left(x_{i}\right)$ is the predicted average value of x at i position, $Z(x_{i})$ is the observed value of x at i position, n is the number of spots, A is the cumulative distribution function (CDF) of the real value, F is the predicted cumulative distribution function of the model, and 1{x≥y} is the indicator function, when x≥y the value is 1, otherwise it is 0.

#### 2.4.3 BO algorithm

In the process of parameter adjustment, the objective function is unknown and nonconvex, resulting in a huge amount of calculation and a poor solution. In order to solve such problems, the BO algorithm is introduced. The KNN, RFR, and GBRT (Sun et al., 2021; Zhang et al., 2021) models are used as the initial model of the chlorophyll content of *D. giganteus*, and the BO algorithm is used to optimize the machine learning model. Its core idea is to use prior knowledge to approximate the posterior distribution of the unknown objective function and then select the next sampling hyperparameter combination according to the distribution (Zhang et al., 2021). The probability model is used to represent the complex black box function, which makes the model more accurately meet the behavior of the black box function, effectively reduces the unnecessary evaluation of the objective function, and theoretically guarantees the final convergence to the global optimal solution (Cui and Yang, 2018; Zhou et al., 2023), so as to reduce the model calculation amount and optimize the objective model parameters, thereby improving the model estimation accuracy.

**Figure 4:**
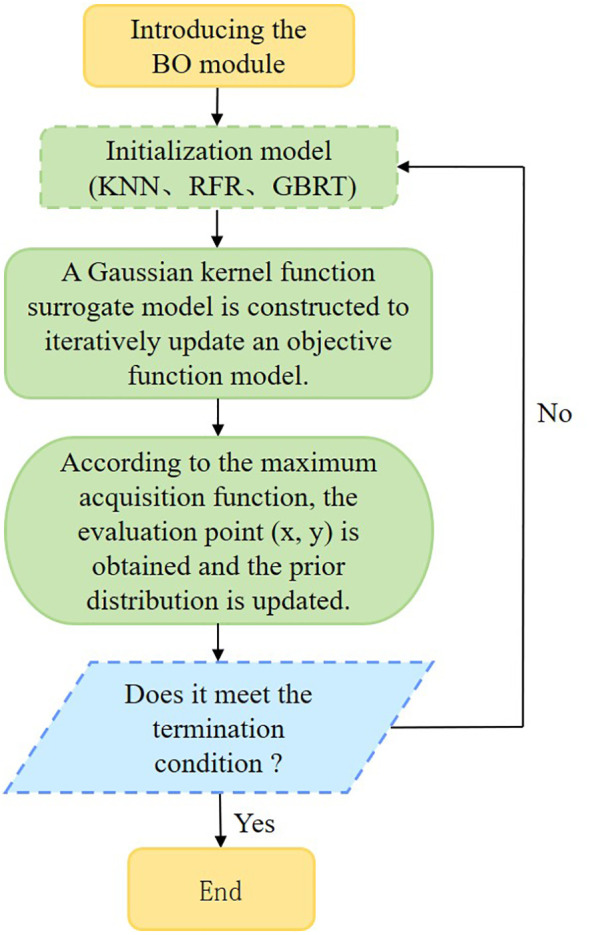
The algorithmic flow of BO for KNN, RFR, and GBRT models.


This study primarily optimizes the key parameters of KNN, RFR, and GBRT models through 2,000 iterations to identify the optimal parameters for modeling. The definitions of each optimized parameter (Zhang et al., 2021) are presented in **Table 6**. BO is an iterative process consisting of six steps (**Figure 4**), which include three core steps: (1) The next most potential evaluation point $x_{t}=argmax_{x\in X}\alpha (x|D_{1:t-1})$ is selected according to the maximum acquisition function. (2) Calculate the objective function value $y_{\text{t}}=\theta \left(x_{t}\right)+\epsilon_{t}$ according to the selected evaluation point $x_{t}$ (3) The newly obtained input-observation pair $\left\{x_{t},y_{t}\right\}$ is added to the historical observation set $D_{1:t-1}$ and the probabilistic surrogate model is continuously updated to prepare for the next model iteration. In order to find the best number of simulation optimizations and the best model parameters for modeling, the optimization process uses the Bayes theorem (Cui and Yang, 2018):

**Table 6:** Description of KNN, RFR, and GBRT model parameters.

Model	Parameters	Description	Type
KNN	n_neighbors	The number of neighbors to use by default for neighbor’s queries.	int
	weights	The weight function used in the prediction.	Str or callable
RFR, GBRT	max_depth	The maximum depth of the tree.	int
	n_estimators	The number of trees in the forest.	int
	min_samples_split	The minimum number of samples required to split an internal node.	int or float
	min_samples_leaf	The minimum number of samples required to be at a leaf node.	int or float

$$\begin{array}{c} \;\;\;\text{p}\left(\text{θ}|{\text{D}}_{1:\text{t}}\right)=\frac{\text{p}\left({\text{D}}_{1:\text{t}}|\text{θ}\right)\text{p}\left(\text{θ}\right)}{\text{p}\left({\text{D}}_{1:\text{t}}\right)}\; \end{array}$$

Where θ represents the initial parameters, the observed set is denoted by $D_{1:t}=\left\{\left(x_{1},y_{1}\right),\left(x_{2},y_{2}\right),\ldots ,\left(x_{t},y_{t}\right)\right\}$ the decision vector is denoted by $x_{1}$ the observed value is denoted by $y_{1}=\theta \left(x_{t}\right)+\epsilon_{t}$ and the observation error is denoted by $\epsilon_{t}$ . The likelihood distribution of y is denoted by $p\left(D_{1:t}|\theta \right)$ and the posterior probability distribution of θ is denoted by p(θ) . The marginal likelihood distribution of the marginalized θ , primarily used for hyperparameters in Bayesian analysis, is denoted by $p\left(D_{1:t}\right)$ . The posterior probability distribution of θ is denoted by $p\left(\theta |D_{1:t}\right)$ .

### 2.5 Accuracy evaluation of the model

In this study, leave-one-out cross-validation (LOOCV) was used to verify the prediction accuracy of the chlorophyll content RS estimation model and the accuracy of its estimation results. For small sample data, LOOCV was applied to iteratively train and validate the model, addressing issues related to using the same training and validation sets and helping to avoid local optima in model fitting (Song et al., 2022; Zhou et al., 2023, 2024). Compared with K-fold cross-validation, the results of this verification method have higher reproducibility and are not affected by random factors, so it has stronger robustness (Song et al., 2022; Xia et al., 2024). At the same time, it can also effectively solve the overfitting or underfitting problem of the model. In the RS estimation model of chlorophyll content of *D. giganteus*, comprehensive evaluation indexes, including *R* ^2^, RMSE, rRMSE, and overall prediction accuracy (*P*), were used. A higher value of *R^2^* indicates a better fit of the model. Conversely, lower values of RMSE and rRMSE, along with a higher *P* value, suggest increased accuracy of the model. Therefore, achieving values close to 1 for *R^2^* and larger values for *P*, while minimizing RMSE and rRMSE, are indicative of better model accuracy. Conversely, suboptimal values of these metrics indicate a less ideal model fit. The evaluation metrics are defined by the following formulas:


$${\text{R}}^{2}=\frac{\sum_{\text{i}=1}^{\text{n}}({\hat{\text{y}}}_{\text{i}}-\bar{\text{y}}{)}^{2}}{\sum_{\text{i}=1}^{\text{N}}({\text{y}}_{\text{i}}-\bar{\text{y}}{)}^{2}}$$

$$\text{RMSE}=\sqrt{\frac{{\sum_{\text{i}=1}^{\text{n}}\left(9{\text{y}}_{\text{i}}-{\hat{\text{y}}}_{\text{i}}\right)}^{2}}{\text{n}}}$$

$$\text{rRMSE}=\frac{\text{RMSE}}{\bar{\text{y}}}$$

$$\text{P}=\left(1-\frac{\text{RMSE}}{\bar{\text{y}}}\right)$$

Where n is the number of samples, ${\hat{y}}_{i}$ is the predicted value of the model, $y_{i}$ is the measured value of chlorophyll content, and $\bar{y}$ is the predicted average value of the model, $\bar{y}=\frac{1}{n}\sum_{i=1}^{n}y_{i}\;$ (Xia et al., 2024).

## 3 Results and analysis

### 3.1 Accuracy evaluation of the model

**Figure 5:**
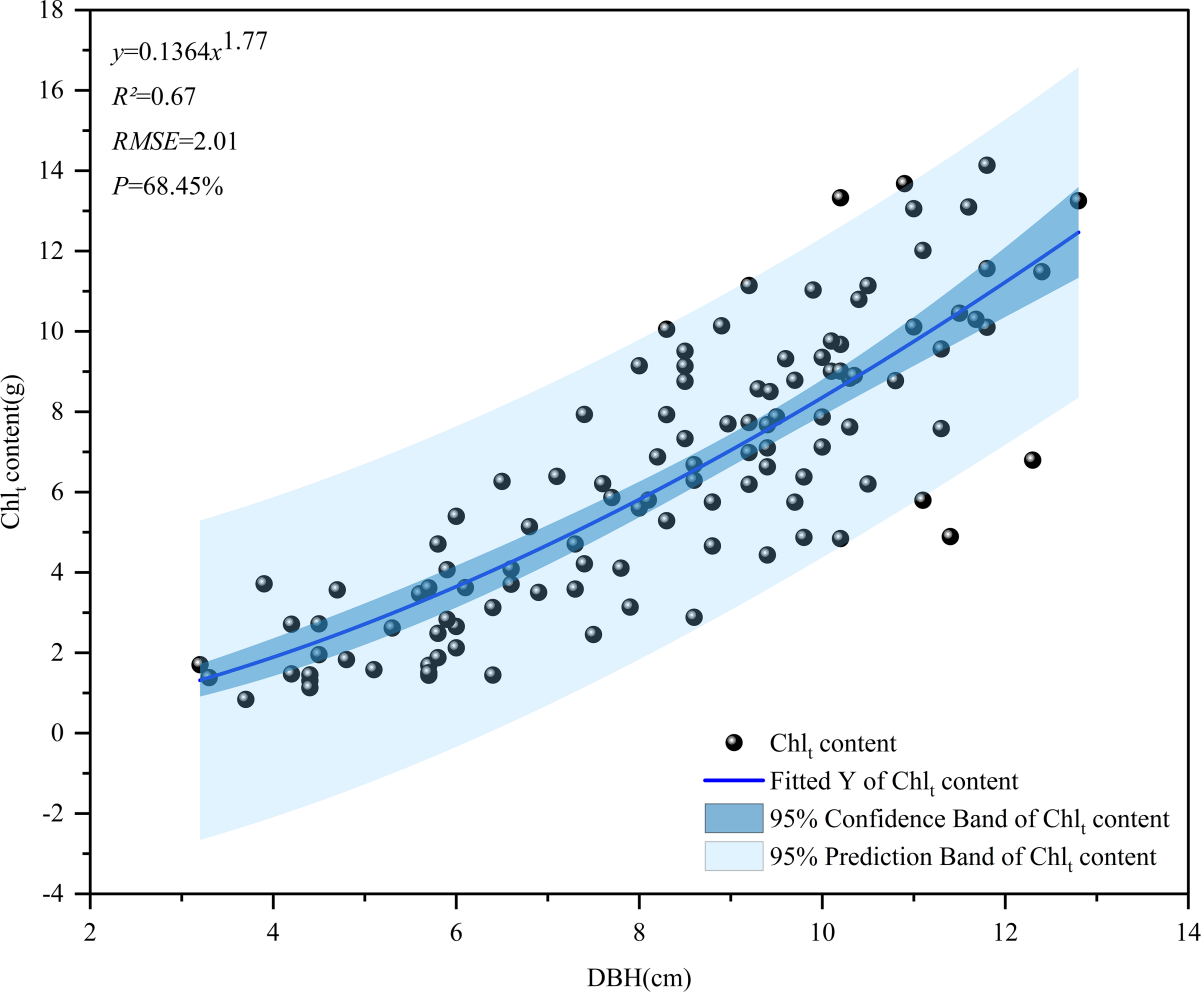
Single plant chlorophyll content model of *D. giganteus*.

In this study, a basic model was created to explore the relationship between the chlorophyll content per plant of *D. giganteus* and the DBH per plant. The independent variable in the model was the DBH per plant, while the chlorophyll content per plant of *D. giganteus* served as the dependent variable (**Figure 5**). A power function was employed as the noncurve fitting function, with a confidence interval set at 95%. Analysis of the fitting curve revealed that the predictive variable values closely aligned with the upper or lower limits of the confidence interval. It can be seen from the *R* ^2^ value of the model fitting evaluation index that the model has a good fitting degree and stable performance. It can be seen from the RMSE and *P* values that the accuracy of this model is similar to that of the single-plant model established by Xia et al. (2024). Furthermore, a positive correlation was established between the chlorophyll content of individual plants and their respective DBH. This implies that, under specific growth conditions, an increase in the DBH corresponds to a rise in chlorophyll content, reflecting the natural growth patterns of *D. giganteus*.

### 3.2 The regression results of GEDI characteristic parameters

**Figure 6:**
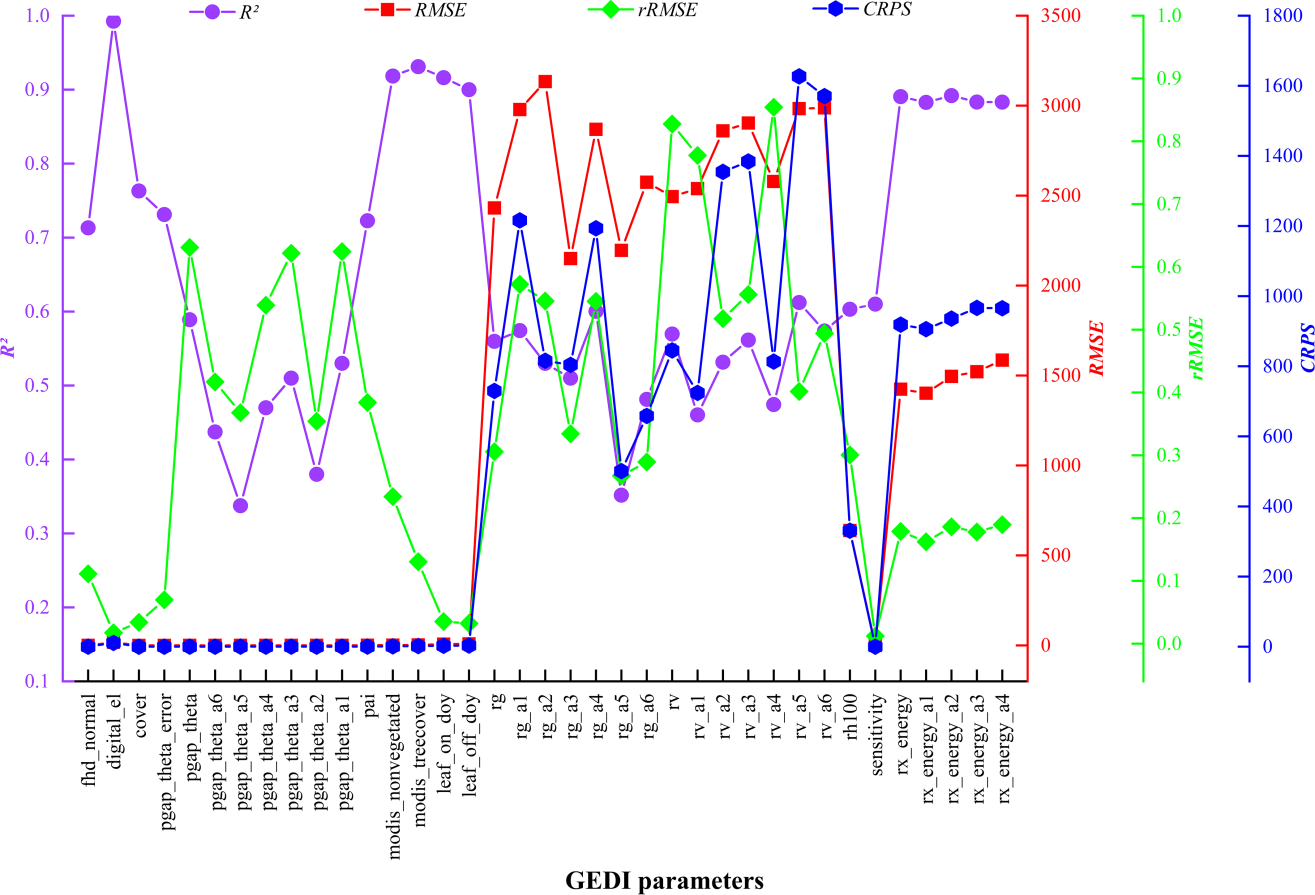
GEDI characteristic parameters and evaluation indexes based on the EBKRP method.

The EBKRP method was utilized in this study to forecast the unknown spatial distribution of GEDI characteristic parameters within the study area. It can be seen from **Figure 6** that the prediction accuracy *R* ^2^ of 37 parameters was 0.34~0.99, RMSE was 0.012~3,134.005, rRMSE was 0.011~0.854, and CRPS was 965.492~1,626.887. Among them, the spatial regression prediction results of digital_el and modis_treecover parameters are the best, the prediction accuracy of rx_energy series parameters is good, the estimation accuracy of single parameters such as modis_nonvegetated, leaf_on_doy, leaf_off_doy and series parameters such as rg and rv is high, and the prediction accuracy of pgap_theta series parameters is the lowest. In general, the estimation accuracy and prediction results vary significantly across different types of parameters, whereas the prediction results for the same type of parameters are similar. The closer the characteristic parameters are to the covariate vegetation index and terrain factors, the higher the prediction accuracy. Therefore, it shows that the EBKRP method respects the basic attributes of the initial measured data values. The data distribution between different types of parameters is relatively discrete, while the data distribution of the same type is relatively concentrated, reflecting the volatility of data distribution.

### 3.3 Characteristic variable selection results

**Figure 7:**
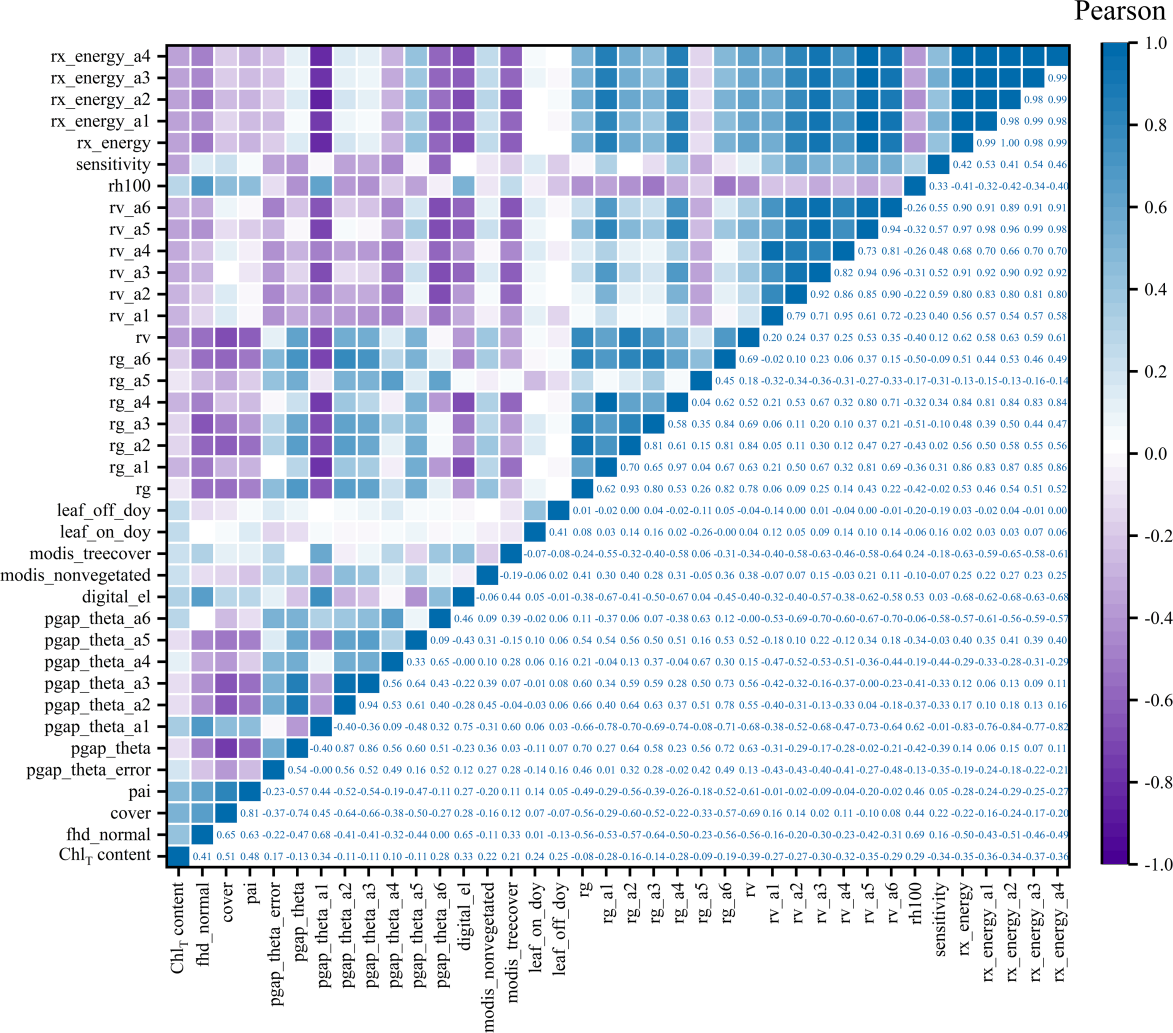
The correlation matrix between GEDI parameters and chlorophyll content.

In order to analyze the interaction between GEDI characteristic variables and chlorophyll content, this study used Pearson (the absolute value of the correlation coefficient is between 0 and 1, the closer to 1, the stronger the correlation, and vice versa) and RF (through a large number of decision trees to obtain the importance of characteristic variable factors for comprehensive scoring ranking) two methods to select the optimal characteristic variable factors as modeling parameters. According to **Figure 7**, the parameters with strong correlation were selected as the independent variable factors for RS estimation of chlorophyll content. Among the 37 parameters, the Pearson correlation level was set to be significant at the 0.05 level, and the absolute value of the correlation coefficient was 0.079~0.509. To comprehensively consider factors such as prediction accuracy requirements, sample size, and model interpretability, the study set a correlation threshold of greater than 0.37 and selected five characteristic variables as the optimal modeling variables. The top five characteristic variable factors of the correlation from high to low are cover, pai, fhd_normal, rv, rx_energy_a3, and the correlation coefficients are 0.51, 0.48, 0.41, − 0.39, and − 0.37, respectively.

**Figure 8:**
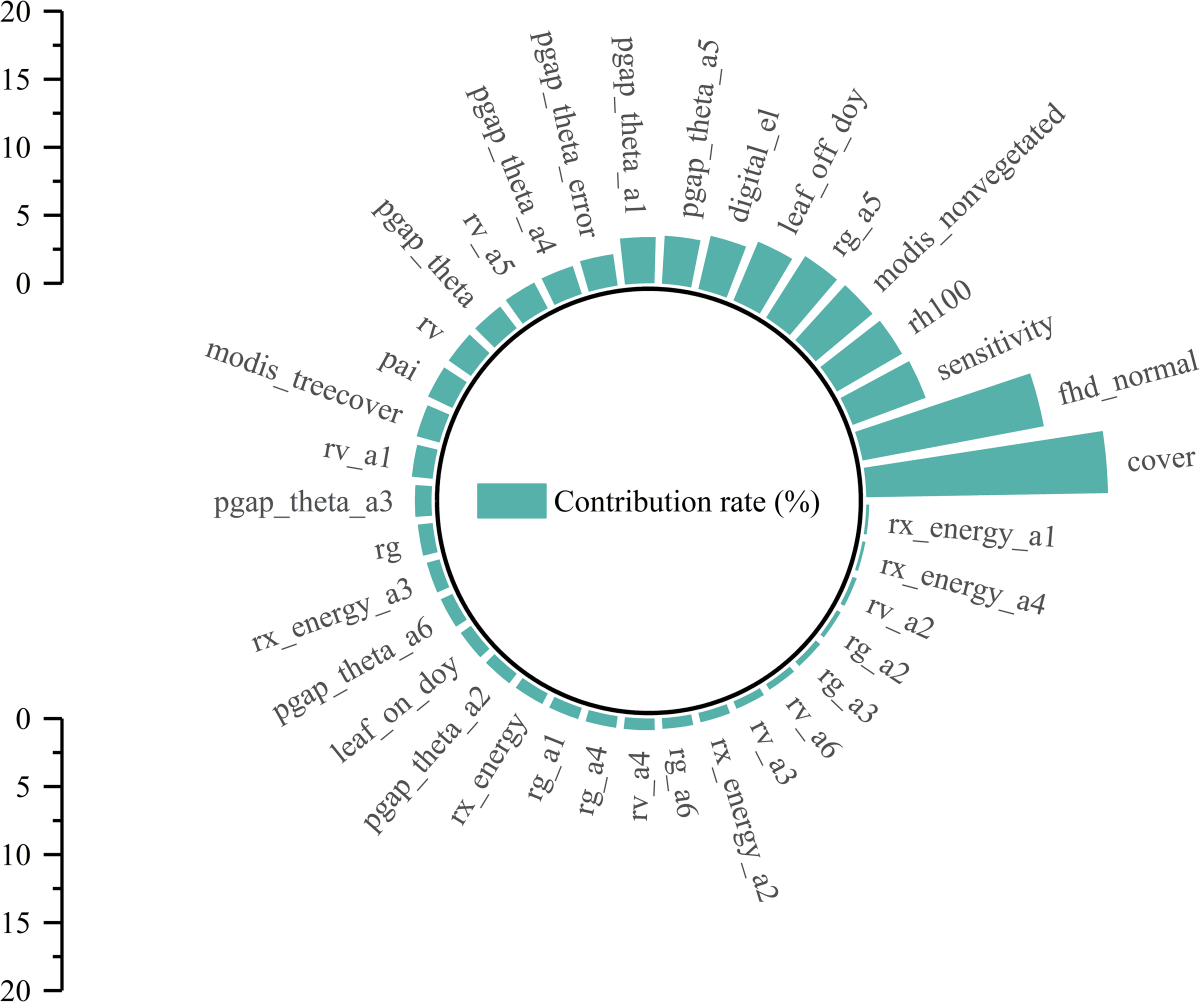
The feature importance contribution ratio of GEDI modeling parameters.

In addition, the 37 GEDI parameters extracted in this study were sorted and evaluated by RF. **Figure 8** shows that the contribution is 0.21%~17.19%. In order to select high-quality RS modeling parameters, an importance greater than 5.5% was set as the threshold, and a total of 5 high-quality parameters were selected. The feature importance from high to low is cover, fhd_normal, sensitivity, rh100, and modis_nonvegetated. The contribution rates were 17.79%, 13.58%, 5.73%, 5.54%, and 5.47%, respectively.

In summary, using two different methods to screen the GEDI feature variables, it is found that the best modeling parameters selected by different methods are also different. However, in this study, the two parameters of cover and fhd_normal are the common independent variables of RS modeling. Specifically, the cover parameter indicates the total vegetation coverage. Given that chlorophyll mainly exists within leaves, higher coverage implies more vegetation and leaves, thereby accommodating more chlorophyll. The fhd_normal, a leaf-height diversity index, reflects the structural complexity of the canopy in the vertical direction and thus indirectly influences the chlorophyll content. Vegetation at different heights and levels experiences variations in environmental conditions such as light, temperature, and humidity. Typically, the leaves in the upper part of the canopy receive more light, motivating plants to synthesize more chlorophyll for photosynthesis. In contrast, the leaves in the lower canopy have a lower chlorophyll content due to light limitation. Consequently, in the modeling process, the cover serves as a macroscopic and intuitive measure of chlorophyll content, while fhd_normal, which reflects the complexity of the vertical structure of the canopy and indirectly affects chlorophyll content, can be regarded as an important independent variable for predicting regional chlorophyll content.

### 3.4 Results of RS Modeling

**Figure 9:**
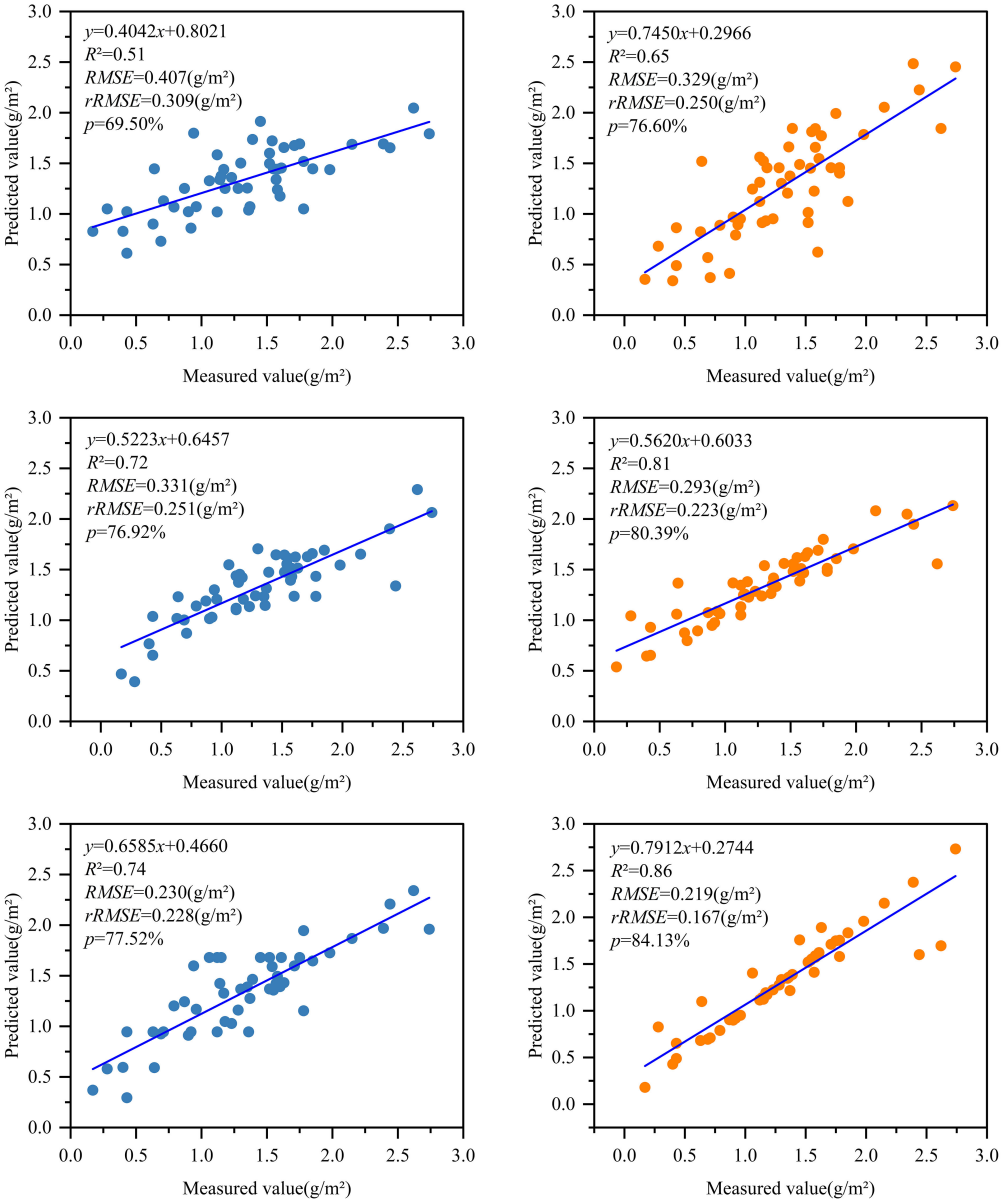
The results of the BO algorithm model construction using Pearson and RF method selection variables. Note: Horizontally, BO-KNN, BO-RFR, and BO-GBRT are arranged from top to bottom, while vertically, Pearson and RF are organized from left to right.

After optimizing the independent variable factors of two groups of RS modeling of different chlorophyll content by Pearson and RF methods, the BO-KNN, BO-RFR, and BO-GBRT three algorithm models were used to develop the best RS estimation model of chlorophyll content of D. giganteus at regional scale. The parameter modeling optimized by the Pearson method found that after 2,000 times of model kernel parameter optimization, 1,000, 500, and 1,000 times were finally determined as the best optimization times of BO-KNN, BO-RFR, and BO-GBRT models, respectively. The parameter modeling optimized by the RF method found that after 2,000 times of model kernel parameter optimization, 600, 700, and 1,100 times were finally determined as the best optimization times of BO-KNN, BO-RFR, and BO-GBRT models, respectively. The optimal number of iterations for each model is determined by observing a smooth convergence trend after continuous iterations and improvements on the original basic model. This process ensures the model avoids underfitting and overfitting, ultimately yielding the optimal solution. It can be seen from **Table 7** and **Figure 9** that the estimation accuracy *R* ^2^ of KNN, RFR, GBRT, and the model before optimization is 0.30~0.46, RMSE is 0.387~0.512 g/m^2^, rRMSE is 0.291~0.396 g/m^2^, and *P* is 63.26%~74.09%, indicating that although LOOCV is more stable than K-fold cross-validation, the estimation accuracy of the model is not high and needs to be further improved. After optimization, the estimation accuracy *R* ^2^ of BO-KNN, BO-RFR, and BO-GBRT models was 0.51~0.86, RMSE was 0.219~0.407 g/m^2^, rRMSE was 0.167~0.309 g/m^2^, and *P* was 69.50%~84.13%. Compared with the model before optimization, the accuracy of the optimized model is effectively improved, and the accuracy of the prediction results is enhanced.

**Table 7:** Results of RS initial estimation model for chlorophyll content of *D. giganteus*.

Regression model	Modeling parameter optimization method	*R*²	RMSE	rRMSE	*P* (%)
KNN	Pearson	0.30	0.511	0.396	62.36
	RF	0.37	0.452	0.340	69.60
RFR	Pearson	0.35	0.495	0.376	65.43
	RF	0.41	0.445	0.313	70.86
GBRT	Pearson	0.37	0.454	0.340	68.99
	RF	0.46	0.387	0.291	74.09

Combined with **Table 7** and **Figure 9**, from a vertical perspective, the estimation accuracy of the best variables selected by different models for the same feature variable selection method is quite different, while the improved regression model based on the BO algorithm has higher estimation accuracy than the initial regression model, and the predicted chlorophyll content is more accurate. Among them, the BO-GBRT model has the best overall performance. From a horizontal perspective, the same model shows slight differences in accuracy when using the best variables selected by different feature variable selection methods. Notably, the feature variables selected by the RF method are more accurate than those selected by the traditional Pearson method. Therefore, the BO-GBRT model established using the five best characteristic variables selected by the RF method was ultimately chosen as the best RS estimation model for the chlorophyll content of *D. giganteus* in the study area. The maximum *R²* of the model was 0.86, the minimum RMSE was 0.220 g/m², the minimum rRMSE was 0.167 g/m², and the maximum *P* was 84.13%.

### 3.5 Regional-scale distribution of chlorophyll content in *D. giganteus*

**Figure 10:**
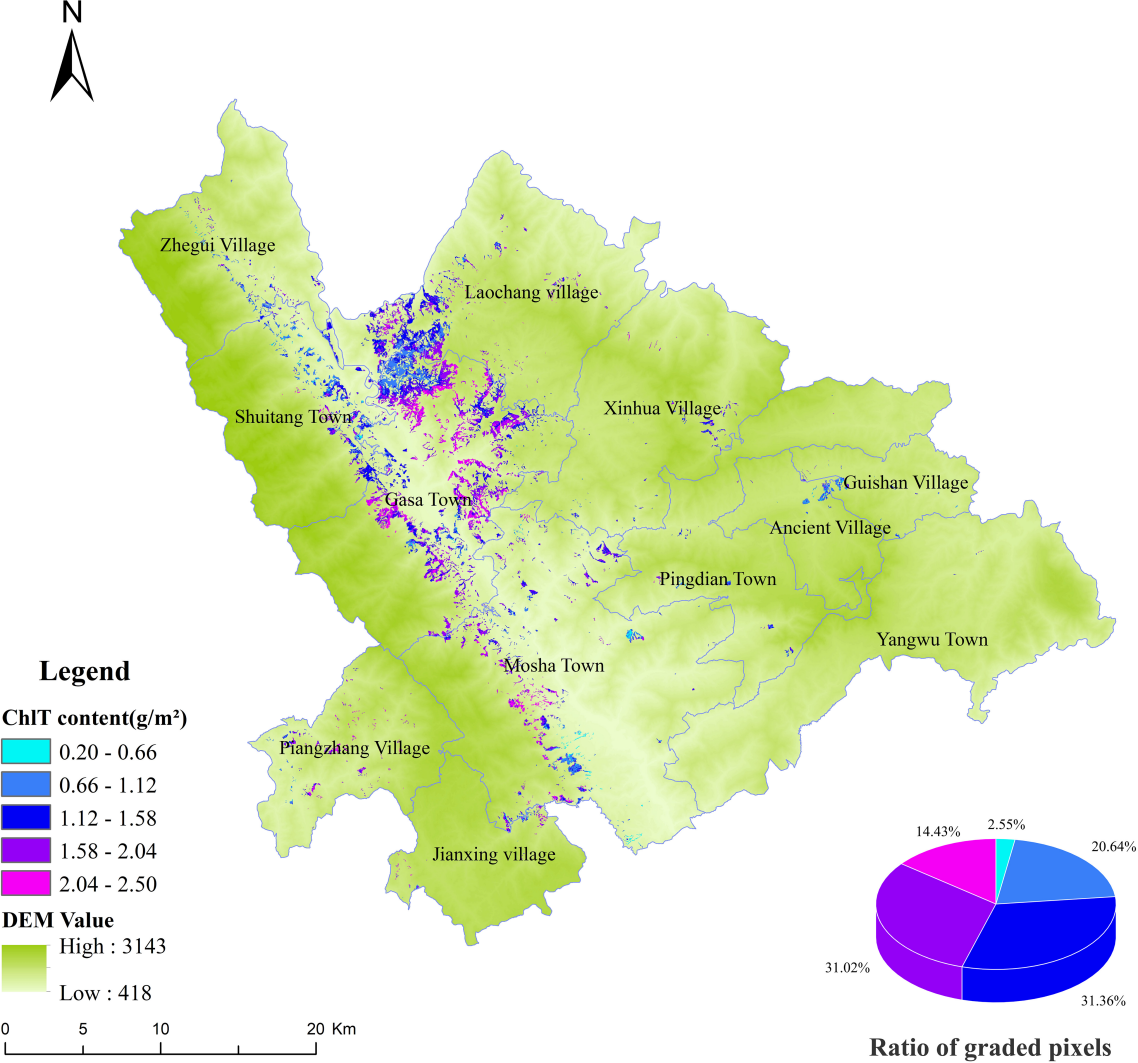
Spatial distribution of chlorophyll content in the study area for *D. giganteus*.

In this study, the best BO-GBRT model was selected to predict the spatial distribution of chlorophyll content in the study area (**Figure 10**). The chlorophyll content of *D. giganteus* ranges from 0.20 g/m² to 2.50 g/m², with an average of approximately 1.44 g/m². The maximum chlorophyll content is 2.50 g/m², while the minimum is 0.20 g/m². Throughout the study area, regions of high and low chlorophyll content are interspersed, indicating significant regional differences. Areas of high chlorophyll content are mainly concentrated at the junctions of Shuitang Town, Laochang Township, and Gasa Town, as well as near the Gasa River and the western region of Mosha. Conversely, the eastern region, which has a higher population density and fewer *D. giganteus* distributions, exhibits lower chlorophyll content. Additionally, the spatial distribution map indicates that the chlorophyll content of *D. giganteus* is primarily concentrated between 1.12 g/m² and 1.58 g/m². This range has the highest number and proportion of pixels, reflecting the favorable growth conditions of *D. giganteus* in the study area. This distribution pattern may be related to the ecological habits and environmental factors of *D. bambusoides*. It was found that the altitude of the western region coincides with the optimal growth altitude of *D. giganteus* (300~1,200 m), while the eastern region does not. Future studies should include factors such as light, temperature, precipitation, and soil nutrients to further analyze the interaction between chlorophyll content and ecological or environmental factors.

## 4 Discussion

This study introduces a novel method for converting point attribute data into area attribute data to estimate the chlorophyll content of *D. giganteus*. A BO algorithm was employed to refine the initial machine learning regression model, thereby enhancing estimation accuracy. First, a relative growth equation model for the chlorophyll content of individual *D. giganteus* plants was established and subsequently used to estimate the chlorophyll content of 52 sample plots. Finally, by applying the BO machine learning model, the chlorophyll content of Xinping County was successfully estimated, achieving the goals of reducing costs, improving efficiency, and enhancing accuracy. In this study, spaceborne LiDAR data were used as the dependent variable, combined with optical RS data as explanatory variables to estimate vegetation chlorophyll content. The main challenge lies in addressing the discreteness of GEDI spots and explaining the relationship between optical RS data from Sentinel-2 and DEM-extracted variable factors with GEDI feature parameters. Therefore, this study aims to tackle these challenges to improve the accuracy and precision of estimating the chlorophyll content of *D. giganteus*. By tackling the aforementioned issues, the focus is on exploring the ability of the EBKRP method to improve the accuracy and precision of regression prediction results after converting GEDI point data into area data. Additionally, the potential of the BO algorithm in RS modeling and inversion of chlorophyll content at the county scale is evaluated, providing a reference for medium- and large-regional-scale chlorophyll content inversion.

### 4.1 Analysis of EBKRP results

To efficiently, cost-effectively, and accurately obtain the area attribute information of GEDI feature parameters, this study employed the EBKRP method. The EBKRP method involves determining fixed covariates (such as vegetation index and terrain factor) as explanatory factors for the main variables (GEDI parameters), which improves the prediction accuracy of GEDI parameters in unknown spatial distributions and to strengthens their relationship with chlorophyll content. This method overcomes the discontinuity of GEDI spot data distribution and provides a new research perspective for the RS estimation of vegetation chlorophyll content at medium and large regional scales. Additionally, the results of this study showed that the accuracy of each GEDI index predicted by the EBKRP method was high; the *R*² values ranged from 0.34 to 0.99, RMSE values from 0.012 to 3,134.005, rRMSE values from 0.011 to 0.854, and CRPS values from 965.492 to 1,626.887. This significantly reduced error transfer and was consistent with the findings of Giustini et al. (2019) and Lötter and Le Maitre (2021), laying a solid foundation for the accurate estimation of chlorophyll content in the study area. Compared with the results of Xu et al. (2023) and Zhou et al. (2023), this method not only surpasses OK interpolation in prediction accuracy but also produces GEDI parameter maps with smooth characteristics and without obvious striping effects (strip effects refer to regular strips or ripples in the images of Kriging interpolation or Kriging regression results). This indicates that as prediction accuracy increases, the striping effect gradually weakens, which aligns with the First Law of Geography (spatial autocorrelation), the Second Law (spatial heterogeneity), and the Third Law (geographical similarity) (Zhu et al., 2020). When comparing the prediction accuracy of different spatial interpolation or spatial regression methods, researchers can use ANUSPLIN software (Guo et al., 2020), the Bayesian Maximum Entropy (BME) method (Christakos and Li, 1998), or the Stepwise Principal Component Logistic Regression-Kriging method (Hengl et al., 2004). To mitigate the striping effect in kriging interpolation, spectral data from the same or adjacent strips can be thinned using geostatistical software like ArcGIS or programming software such as Python, operating at 100-m intervals (Xu et al., 2023). However, it is important to note that this method requires stringent selection criteria for the quality of spot data and is not applicable in studies where spot data are scarce. Data thinning may result in the loss of a large amount of sample data, reducing the accuracy of interpolation and making the estimation results less precise. In contrast, the EBKRP method maintains interpolation accuracy while effectively mitigating the striping effect.

### 4.2 The influence of parameter selection on model accuracy

The rationality of the selection of independent variables is directly associated with the performance of the model, the reliability of the estimation results, and the universality of the model. In this study, the selection of independent variables focuses on those relevant to both the individual plant model and the regional scale estimation model. The selection of independent variables for individual plant models has been rarely addressed in previous studies. However, some scholars have conducted related work in the study of tree biomass and found a significant correlation between tree aboveground biomass and DBH. However, in the process of field measurement, the measurement of tree height often has large errors, so the height is not an ideal modeling parameter (Araújo et al., 1999; Chave et al., 2001). To reduce costs, enhance work efficiency, and avoid errors associated with height measurements that could affect the results, the study suggests that a regression model using DBH as an independent variable can more accurately reflect the aboveground biomass of various bamboo species as DBH changes (Chave et al., 2001; Yang et al., 2008; Ji et al., 2015). Compared with the traditional destructive sampling method, the modeling method based on the relative growth equation has a wider range of general adaptation, which provides an important reference value for the estimation of chlorophyll content and forest health monitoring in the future.

In selecting independent variables for the regional-scale estimation model of *D. giganteus* chlorophyll content, many previous studies have focused on extracting relationships between optical RS image data and measured chlorophyll content samples to estimate chlorophyll content in the study area (Houborg et al., 2015; Ma et al., 2021), while overlooking the rich feature information provided by GEDI L2B product data. Its parameters, such as pai, cover, and fhd_normal, show strong correlations with chlorophyll content and provide better interpretive accuracy. According to the results of Pearson and RF methods, it can be seen that the independent variables and the number of RS modeling variables selected by different methods will be different, but the factors with strong explanatory power and large contributions to modeling will be retained. Compared with the Pearson method, the RS estimation model constructed by the parameters selected by the RF method is more accurate, and the best estimation model (BO-GBRT) of the chlorophyll content of the *D. giganteus* in the study area was explored in the RF. The *R* ^2^ is 0.86, the RMSE is 0.291 g/m^2^, the rRMSE is 0.167 g/m^2^, and the *P* is 84.13%, which may be related to the characteristics of the RF itself. When sorting the importance of features, it can not only ensure randomness but also eliminate data redundancy (Wu et al., 2017), making the RS estimation model more stable and generalized. It also reflects that this parameter selection method is suitable for the BO-GBRT model, and is consistent with the results of Zhou et al (Zhou et al., 2023, 2024). using this method to estimate the forest canopy density in northwest Yunnan, China. In future research, methods such as KNN, GBRT, and Boruta (Zhang et al., 2022) can be introduced to screen the modeling factors. First, the KNN method can be used to calculate the distance correlation index between each potential factor and the target variable, and then we can find the factor combination that is closer to the target variable in the feature space for modeling. Alternatively, the GBRT method can be employed. By iteratively training the decision tree to minimize the loss function, we can identify parameters that contribute more to the prediction of the target variable for modeling. Additionally, the Boruta method can also be utilized to overcome the possible deviation of the RF itself in evaluating the feature importance. Through comprehensively assessing the importance of factors, more superior feature parameters can be selected, thereby improving the accuracy of chlorophyll content modeling and the precision of the estimation results. Additionally, this study confirmed the application of GEDI L2B product data extends beyond studying tree biomass and carbon storage. It can also be used for RS estimation and inversion of chlorophyll content at medium and large.

### 4.3 Model error propagation and the potential of BO algorithm

The selection of sampling methods, the layout of field plots, and the uncertainty of remote sensing estimation models and their parameters may affect the accuracy of remote sensing estimation, thus impacting the precise estimation of chlorophyll content (Qin et al., 2017). The number of modeling samples is closely related to the representativeness of the chosen model. As the number of modeling samples increases, the representativeness of the estimation model increases, and the uncertainty of the estimation correspondingly decreases. However, once a certain threshold is reached, further increasing the number of samples does not significantly enhance the accuracy of the estimation model. Therefore, in order to conserve manpower, material, and financial resources, plots containing *D. giganteus* of different slopes, altitudes, and ages were evenly and randomly established. Meanwhile, to meet the large-sample principle (50) and the accuracy requirements of field investigation (Hua and Zhao, 2021; Song et al., 2022), 52 measured-plot data were investigated in this study for the purpose of modeling research. Based on the inversion results of chlorophyll, it is categorized into five grades, ranging from low to high. The number of samples in each grade is six, 10, 21, nine, and six, respectively, and the proportion of each grade’s pixels to the total pixels is 2.55%, 20.64%, 31.36%, 31.02%, and 14.43%, respectively (**Figure 10**). All these grades are normally distributed, indicating that the sampling is representative and the modeling results are reasonable, thereby reducing the uncertainty and error propagation caused by sampling.

To reduce the inaccuracy of estimation results caused by model error transfer, this study uses the BO algorithm to optimize the hyperparameters of the KNN, RFR, and GBRT models. Under the same non-parametric initial model, the BO algorithm achieves better model estimation accuracy and operates faster compared to PSO, GA, and DE (Cui and Yang, 2018; Cho et al., 2020). The results show that the BO algorithm can significantly improve the prediction accuracy of the machine learning model and make the prediction results more accurate. Compared with before optimization, the *R* ^2^ of the optimized KNN, RFR, and GBRT models increased by 0.34 on average, the RMSE decreased by 0.144 g/m^2^ on average, the rRMSE decreased by 0.093 g/m^2^ on average, and the *P* increased by 8.96% on average. Compared with the method of simulation optimization by fixed parameter times (Zhang et al., 2021; Zhou et al., 2023), this study uses the BO algorithm to search the best simulation optimization times of BO-KNN, BO-RFR, and BO-GBRT models in 2,000 iterations, which significantly improves the prediction accuracy of the model and saves time. The BO-GBRT model (*R*² = 0.86, RMSE = 0.219 g/m², rRMSE = 0.167 g/m², *p* = 84.13%) was selected as the best estimation model for the chlorophyll content in the test area, with values ranging from 0.20 to 2.50 g/m². Currently, there are few studies on the chlorophyll content of bamboo plants, particularly *D. giganteus*. Compared to the RS estimation of total chlorophyll content in wheat leaves by Jin et al. (2012) (*R*² = 0.868, RMSE = 0.384 g/m²), the estimation accuracy of this study is similar. Compared to the studies by Richardson et al. (2002) and Gitelson and Merzlyak (1997), which only examined the chlorophyll content of single leaves in higher plants, this study extrapolated the chlorophyll content of individual plant leaves to the RS estimation of the overall chlorophyll content in the test area. This offers a crucial reference for evaluating forest health and implementing scientific management of forest resources. However, compared to the study by Xia et al. (2024), this study only utilized a single GEDI dataset for modeling. In the future, collaboration with multisource RS data (such as UAV hyperspectral data and high-resolution data) can be pursued for further research. Additionally, if time cost is not considered, it is possible to try to expand the scope to search for more accurate models or to introduce deep forest algorithms to allow small sample data to undergo deep neural network learning to simulate the optimal estimation model (Xia et al., 2022).

### 4.4 Prospects for estimating large-scale chlorophyll content using GEDI data

The observation range of GEDI covers most regions between 51.6° N and 51.6° N (Crockett et al., 2023), which not only meets the needs of chlorophyll content research in small areas and model portability tests for chlorophyll content in different research areas (Zhu et al., 2022) but also provides data for the estimation and inversion of vegetation chlorophyll content on medium and large regional scales or globally. Although the characteristic parameters such as pai, cover, and fhd_normal are related to the chlorophyll content in spaceborne LiDAR GEDI L2B products, the prediction accuracy of unknown parameter points using different Kriging interpolation methods (Zhu and Lin, 2010; Wang et al., 2014; Khouni et al., 2021) remains to be improved due to the discontinuous light spots and large volume of data. To overcome this challenge, this study explores the use of a Kriging regression method (the EBKRP method). GEDI characteristic parameters are used as main variables, while vegetation index and topographic factor serve as explanatory variables to convert GEDI point attribute data into area attribute information. Additionally, this study introduces the BO algorithm to optimize the machine model, improving the prediction accuracy of unknown GEDI parameter points and enhancing the accurate estimation of chlorophyll content. This study opens up new ideas for the real-time, rapid, and accurate estimation of forest biochemical parameters (Xia et al., 2024), forest structure parameters (Potapov et al., 2021; Xu et al., 2024; Zhou et al., 2024), and agricultural quantitative RS (Kacic et al., 2021) using spaceborne LiDAR data (such as GEDI, ICESat-2/ATLS) or representative sampling points (such as the main variable is chlorophyll content or biomass, fixed covariates are soil nutrients, temperature, light, etc.) in the future. It also provides a reference and potential for research on the inversion of forest biochemical parameters, such as chlorophyll content of vegetation, at medium and large scales with high efficiency and low cost. This is of great significance for studying forest health status, ecosystem function, and carbon cycle process, and offers useful data support for ecological environment protection and sustainable development.

## 5 Conclusions

In order to explore the estimation potential of multi-beam LiDAR data with respect to forest biochemical parameters, the following conclusions have been drawn:

1. The GEDI indexes predicted by the EBKRP method exhibit good accuracy and reliable estimation results. The *R*² ranges from 0.34 to 0.99, and the RMSE ranges from 0.012 to 3134.005. Among these, the wide range of RMSE may be attributed to the complexity and heterogeneity of the data itself. For instance, due to the influence of vegetation structure and irregular terrain, the energy signal returned by the laser becomes complex and unstable, thereby increasing the difficulty and error of spatial regression. The rRMSE ranges from 0.011 to 0.854, and the CRPS ranges from 965.492 to 1,626.887, indicating that the EBKRP method has good development potential in predicting unknown spatial distribution information.
2. The relative growth equation can be used not only as the basic model of individual tree biomass but also as the basic model of *D. giganteus* chlorophyll content, demonstrating good modeling accuracy and estimation results (*R*² = 0.67, RMSE = 2.01, *p* = 68.45%).
3. Based on different feature variable selection methods, the selected modeling parameters and results differ. Notably, common parameters such as vegetation coverage (cover) and leaf height diversity index (fhd_normal) are closely related to chlorophyll content. Moreover, the model constructed with parameters selected by the RF (with *R*² ranging from 0.65 to 0.86) performs better than the model constructed with parameters selected by the Pearson correlation coefficient method (with *R*² ranging from 0.51 to 0.74).
4. The utilization of the BO algorithm to optimize the key parameters of the machine learning model significantly enhances the accuracy of the RS estimation model. The *R* ^2^ of the optimized machine learning models BO-KNN, BO-RFR, and BO-GBRT increased by an average of 0.34, RMSE decreased by an average of 0.144 g/m^2^, rRMSE decreased by an average of 0.093 g/m^2^, and *P* increased by an average of 8.96%. This greatly reduces the model error transfer, making the RS estimation results for the chlorophyll content of *D. giganteus* more reliable. Therefore, GEDI data, in addition to its application to forest structure parameters like biomass, canopy closure, and canopy height, is also feasible and reliable for estimating forest biochemical parameters. This not only lays the foundation for global forest ecosystem monitoring research but also advances the application and development of RS technology.

## Data Availability

The original contributions presented in the study are included in the article/supplementary material. Further inquiries can be directed to the corresponding author.
